# Self-polymerized platinum (II)-Polydopamine nanomedicines for photo-chemotherapy of bladder Cancer favoring antitumor immune responses

**DOI:** 10.1186/s12951-023-01993-1

**Published:** 2023-07-22

**Authors:** Ren Mo, Jianati Dawulieti, Ning Chi, Ziping Wu, Zhizhong Yun, Jianjun Du, Xinhua Li, Junfeng Liu, Xiaochun Xie, Kai Xiao, Fangman Chen, Dan Shao, Kewei Ma

**Affiliations:** 1 Department of Urology, Inner Mongolia people’s Hospital, Inner Mongolia Urological Institute, Hohhot, Inner Mongolia 010017 China; 2 School of Biomedical Sciences and Engineering, South China University of Technology, Guangzhou International Campus, Guangzhou, Guangdong 511442 China; 3 National Engineering Research Center for Tissue Restoration and Reconstruction, South China University of Technology, Guangdong, 510006 China; 4 School of Medicine, South China University of Technology, Guangzhou, Guangdong 510006 China; 5 Guangdong Provincial Key Laboratory of Biomedical Engineering Key Laboratory of Biomedical Materials and Engineering of the Ministry of Education, South China University of Technology, Guangzhou, Guangdong 510006 China; 6 Department of Urology, Hohhot First Hospital, Hohhot, Inner Mongolia 010020 China

**Keywords:** Cisplatin, Polydopamine, Bladder cancer, Photochemotherapy, Self-assembly

## Abstract

Systemic administration of platinum-based drugs has obvious limitations in the treatment of advanced bladder cancer (BC) owing to lower tumor accumulation and uncontrolled release of chemotherapeutics. There is an urgent need for advanced strategies to overcome the current limitations of platinum-based chemotherapy, to achieve maximal therapeutic outcomes with reduced side effects. In this study, self-polymerized platinum (II)-polydopamine nanocomplexes (PtPDs) were tailored for efficient chemo-photoimmunotherapy of BC. PtPDs with high Pt loading content (11.3%) were degradable under the combination of a reductive tumor microenvironment and near-infrared (NIR) light irradiation, thus controlling the release of Pt ions to achieve efficient chemotherapy. In addition, polydopamine promoted stronger photothermal effects to supplement platinum-based chemotherapy. Consequently, PtPDs provided effective chemo-photothermal therapy of MB49 BC in vitro and in vivo, strengthening the immunogenic cell death (ICD) effect and robust anti-tumoral immunity response. When combined with a PD-1 checkpoint blockade, PtPD-based photochemotherapy evoked systemic immune responses that completely suppressed primary and distant tumor growth without inducing systemic toxicities. Our work provides a highly versatile approach through metal-dopamine self-polymerization for the precise delivery of metal-based chemotherapeutic drugs, and may serve as a promising nanomedicine for efficient and safe platinum-based chemotherapy for BC.

**Supplementary Information:**

The online version contains supplementary material available at 10.1186/s12951-023-01993-1.

## Introduction

BC is a common cancer of the urinary system, with high prevalence and global incidence [1, 2]. Although surgery and chemotherapy have been considered the primary management options for advanced BC, they are of limited effectiveness in cases of recurrent disease or metastasis, [2–4] in which combination therapy shows benefits in controlling disease progression [3, 5]. With novel therapeutic advances in the field of immunotherapy, renewed focus on BC subtypes has emerged as a realistic alternative to platinum(Pt)-based chemotherapy [6, 7]. Moreover, integrating chemotherapy and immunotherapy appears to be a rational strategy to address the therapeutic gap in patients with BC [8, 9]. Cisplatin is a traditional first-line drug for the chemotherapy of BC, especially in patients with advanced BC [10–12]. However, the therapeutic efficacy of cisplatin is often compromised owing to severe systemic side effects, resulting in high recurrence rates, progression, and both primary and acquired drug resistance [13–16]. Therefore, new strategies are needed to overcome the current limitations of platinum-based chemotherapy, to achieve maximal therapeutic outcomes with reduced side effects.

Advances in nanotechnology provide a unique opportunity to increase the therapeutic performance and reduce the unwanted toxicity of chemotherapy [1, 17–20]. A significant amount of research has been devoted to developing nanocarriers that can release drugs on demand and in response to specific stimuli (such as exogenous light, X-ray and magnetic fields, or endogenous pH and redox) [21–25]. The delivery of platinum drugs to tumor sites has been improved using liposomal or copolymer products in early clinical trials, opening new avenues for efficient and safe platinum-based chemotherapy [26, 27]. Encouragingly, rational designs for sophisticated platinum-based nanoplatforms have been achieved to improve the therapeutic performance of BC with the aid of phototherapy, radiotherapy, or immunotherapy [28–31]. However, these nanomedicines often have complex fabrication processes and make it difficult to load and control the release of platinum drugs and other active agents [26, 32, 33]. These drawbacks, along with a lower combination efficiency, result in unsatisfactory therapeutic performance and unwanted safety profiles. Nanoplatforms incorporating platinum drugs and other active agents are significant but challenging, and they can be released selectively into tumor microenvironments and activated simultaneously by specific stimuli [34, 35]. In this context, nanomedicines built from organic building blocks provide unparalleled properties, such as biocompatibility, biodegradability, and high responsiveness, indicating great potential for clinical translation.

**Scheme 1:**
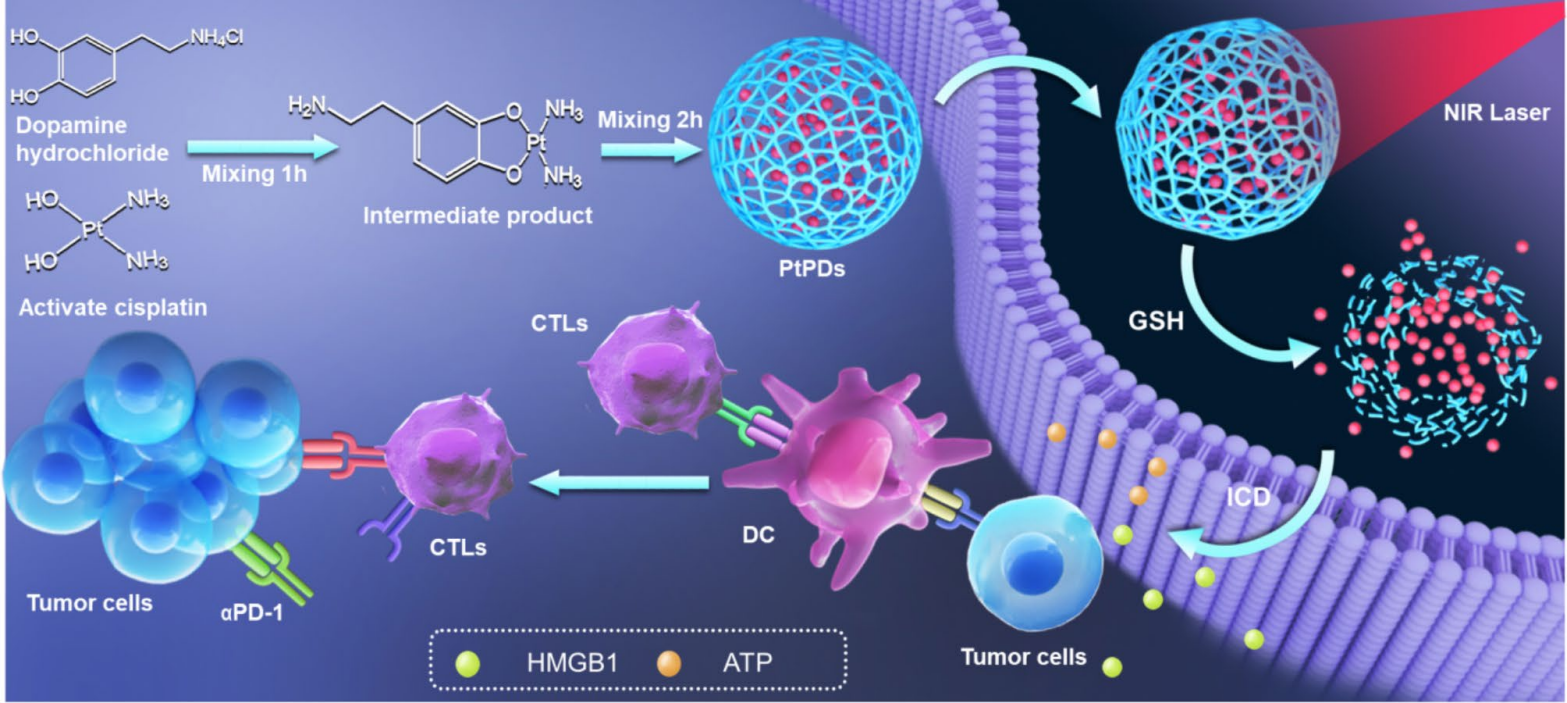
Schematic representation of the synthesis of PtPDs with amplifying ICD effects for chemo-photoimmunotherapy of bladder cancer (BC). ICD: immunogenic cell death; GSH: glutathione; DC: dendritic cells; CTLs: cytotoxic T lymphocyte; HMGB1: High Mobility Group Protein 1; ATP: adenosine triphosphate

Herein, we fabricated a self-assembled nanocomplex containing cisplatin and polydopamine (PDs) for the combination of chemo-photoimmunotherapy against BC (Scheme 1). Platinum (II)-polydopamine nanocomplexes (PtPDs) were self-polymerized between Pt (II) and dopamine molecules. PtPDs with high Pt loading efficiency (11.3%) were disassembled under the combination of a reductive intracellular environment and light irradiation, releasing Pt ions to achieve efficient chemotherapy. PtPDs have emerged as a photothermal (PTT) agent that induces strong phototherapy to supplement platinum-based chemotherapy. Consequently, PtPDs exhibit synergistic chemo-photothermal performance in MB49 BC in vitro and in vivo, strengthening ICD and anti-tumor immunity responses. Combination with a PD-1 checkpoint blockade further evokes systemic immune responses, which completely suppresses tumor growth without inducing systemic toxicities. Our work has developed a promising nanomedicine for highly efficient and safe chemo-photoimmunotherapy of BC. The concept of Pt-dopamine self-polymerization can be extended by conjugation with other metal-based chemotherapeutic drugs, which may aid the rational design of novel nanomedicines to enhance chemotherapy.

## Results and discussions

**Fig. 1:**
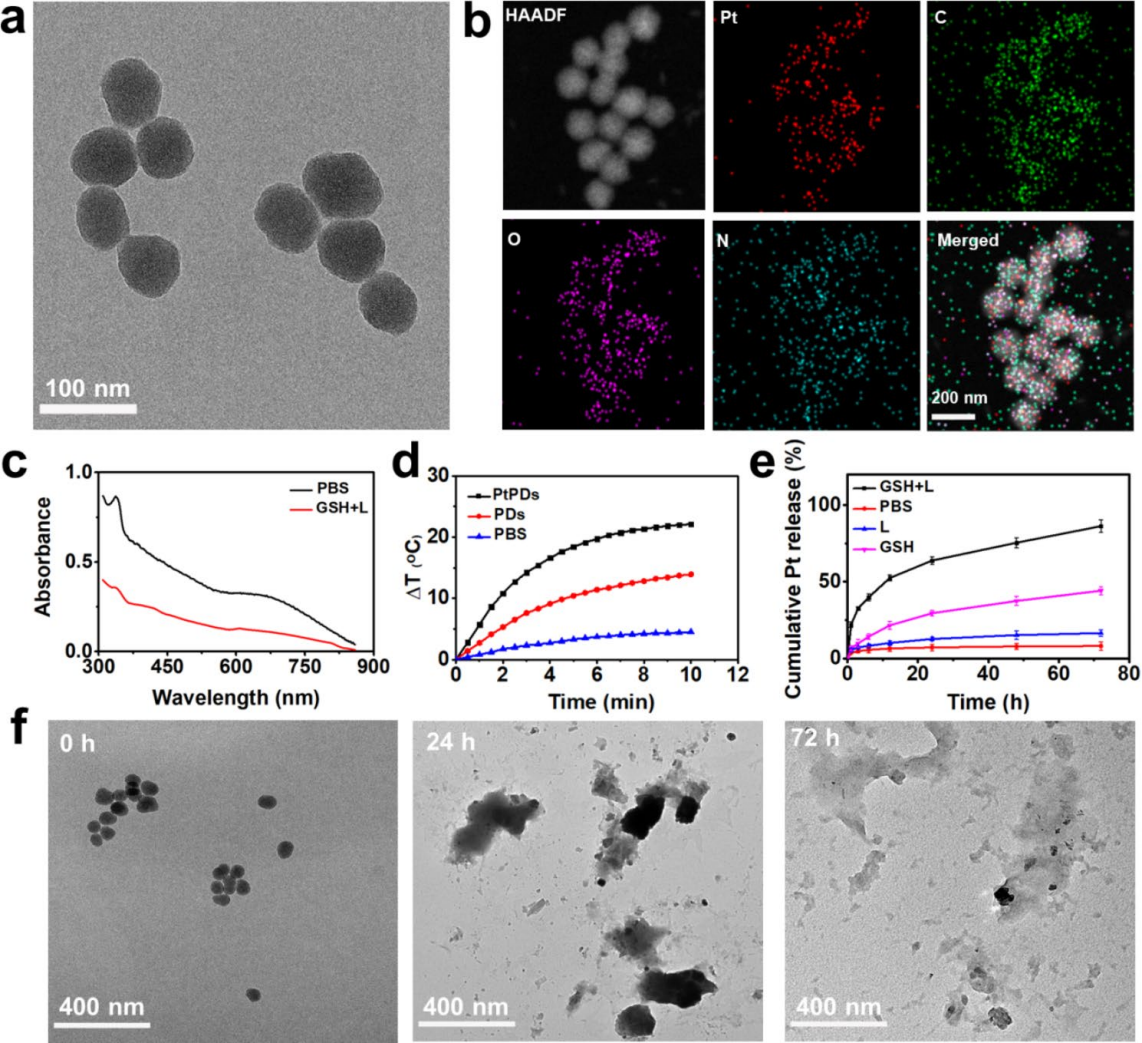
Synthesis and characterization of PtPDs. **(a)** TEM images and **(b)** elemental mapping of PtPDs. **(c)** UV-Vis absorption spectra of PtPDs in physiological (PBS), or reductive GSH (10 mM) environment with 808 NIR light irradiation for 10 min. **(d)** Photothermal capability of different formulations. **(e)** Cumulative Pt release of PtPDs under physiological (PBS), reductive GSH (10 mM) or 808 NIR light irradiation for 10 min. **(f)** TEM images of PtPDs showed degradation in 10 mM GSH upon NIR light irradiation (0.5 W/cm^2^, 10 min)

Self-polymerized PtPDs nanocomplexes were fabricated through a simple one-pot method. As shown in Fig. 1a, Figure S1 and Table S1, the PtPDs exhibited a spherical shape with an average diameter of 67 ± 10.8 nm and a negative charge (-23.1 mV). As shown in Fig. 1b, the presence of Pt, C, N, and O uniformly distributed in PtPDs was demonstrated by elemental mapping. We did not observe significant aggregation of PtPDs in cell culture media after long-term incubation (7 days), indicating good stability by preventing rapid clearance during blood circulation (Figure S1). The UV–vis absorption spectra of the PtPDs in aqueous solution exhibited strong NIR light absorption (Fig. 1c), indicating their potential photothermal capacity. We further evaluated their photothermal properties under NIR laser irradiation. As expected, the temperature of the PtPDs (20 µg/mL) solution elevated by 23.4 °C upon an 808 nm laser light irradiation (0.5 W/cm^2^) for 10 min (Fig. 1d). Interestingly, the photothermal conversion efficiency of the PtPDs was higher than that of the polydopamine nanoparticles (PDs) under the same light irradiation, which might be attributed to the d − d transitions of exogenous platinum ions [36–39]. Pure water, used as a control, showed no measurable temperature elevation. Additionally, the photothermal effect of the PtPDs exhibited concentration-dependent photothermal conversion ability (Figure S2). This photothermal property of PtPDs is sufficient for the hyperthermia or ablation of tumor cells [40–42]. Moreover, photothermal performance without obvious deterioration was observed after five continual irradiation/cooling cycles (Figure S2), indicating good photothermal stability of the PtPDs. Together, these results indicate that the photothermal properties of PtPDs show excellent promise for photothermal therapy.

Because the inclusion of Pt gives PtPDs a robust ability to kill tumor cells, self-polymerized PtPDs with a high Pt loading efficiency of 11.3% (w/w) were detected by inductively coupled plasma-mass spectrometry (ICP-MS). The Pt drug release and disassembly of PtPDs were investigated under reductive conditions, mimicking an intracellular environment. As shown in Fig. 1e, only 4.5% of the Pt drug was released from the PtPDs in PBS, suggesting stability and negligible leakage of the drug during blood circulation. Importantly, PtPDs exhibited a sustainable release of Pt ions (44.2%) at 72 h post-treatment with 10 mM glutathione (GSH), which mimicked the intracellular environment of tumor cells. More importantly, we found that the release rate of Pt ions increased remarkably in the first 12 h under NIR irradiation, and finally reached 86.3% after 72 h in GSH-containing media. In contrast, the light-sensitive release of Pt ions dramatically decreased without GSH exposure, demonstrating that the combination of GSH and NIR irradiation contributed to the release of Pt ions. The GSH-responsive drug release mechanism of PtPDs could be explained by the weakened chelation of metallic complexes owing to the disassembly of polydopamine via the consumption of GSH (Figure S3). The loss of the NIR absorbance peak in the UV − vis spectra indicated the disassembly of PtPDs by creating leakage on the surface or backbone (Fig. 1c) upon light irradiation in the presence of 10 mM GSH, which might facilitate the release of Pt ions. Consistently, transmission electron microscopy (TEM) images further indicated the time-dependent degradation of the PtPDs (Fig. 1f). Collectively, PtPDs efficiently degraded and released Pt ions under the combination of GSH and NIR irradiation, which might enable specific drug release and light-controllable chemotherapy in BC.

**Fig. 2:**
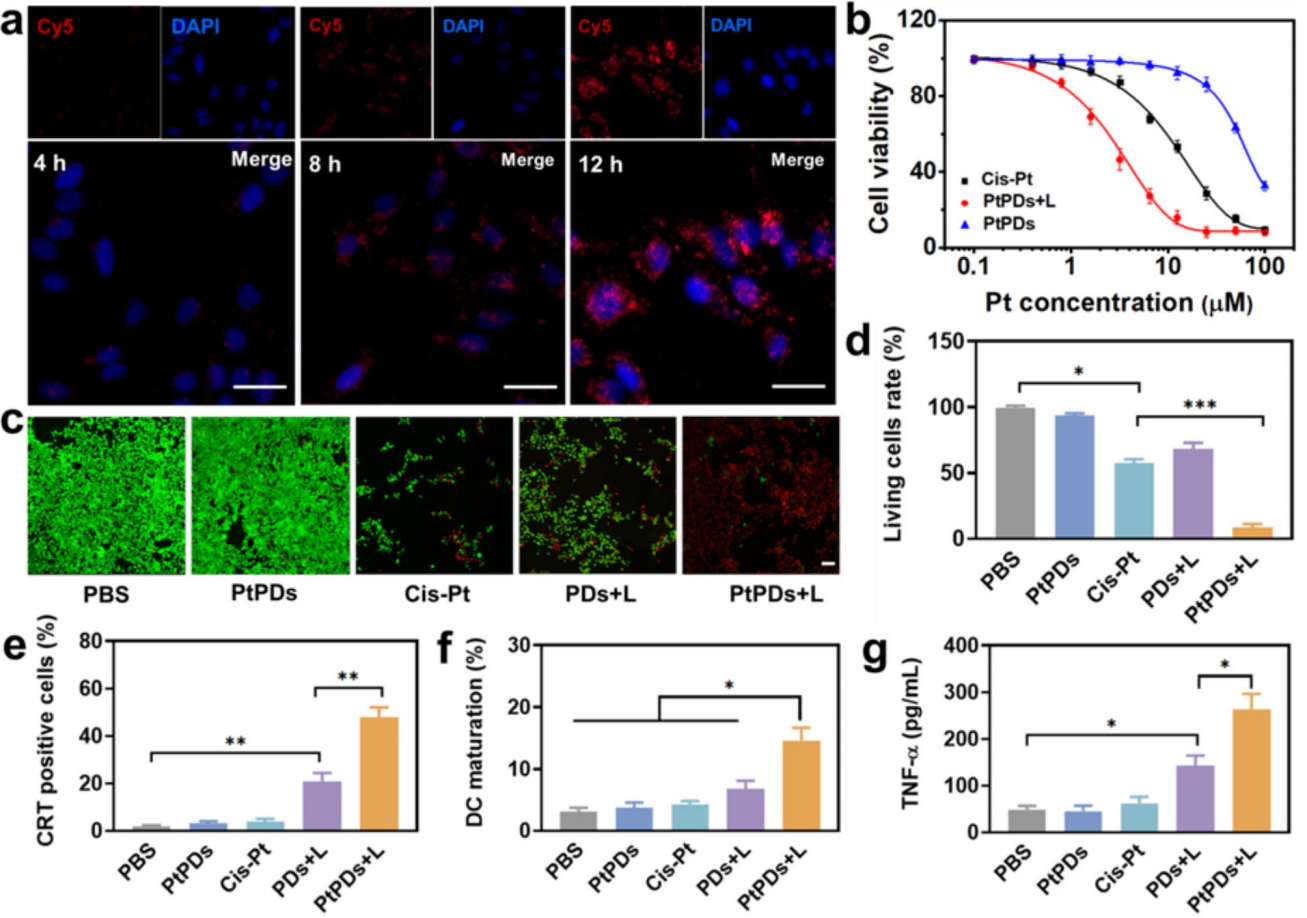
Cell uptake and anti-tumor effect in vitro. **(a)** Confocal laser-scanning microscopy observation of Cy5-labeled PtPDs uptake by MB49 cells. scale bar: 30 μm. **(b)** MB49 cell viability after cultivation for 24 h with different formulations with or without 808 nm laser exposure (0.5 W, 10 min). **(c)** Calcein-AM/PI staining, and **(d)** quantitative analysis of living cell rate in MB49 cells after treating with different formulations. Scale bar: 50 μm. **(e)** Percentage of CRT-positive cells. **(f)** Mature BMDC percentage (CD11c^+^CD40^+^) after co-incubation with treated MB49 cells for 24 h. All results represent the mean ± SD (n = 3). **(g)** The levels of TNF-⍺ in suspension of matured BMDCs. *p < 0.05, **p < 0.01. All results represent the mean ± SD (n = 3)

We next examined whether PtPDs-based PTT and the release of Pt ions affect anti-tumoral therapy. We first studied the cellular uptake of PtPDs by incubating BC cells with dye-labeled nanoparticles. After incubation for 3 h, PtPDs nanoparticles were taken up by cells, and the intracellular accumulation of PtPDs significantly increased after incubation for 8 h (Fig. 2 and Figure S4). The maximum uptake was reached at 12 h, which might be attributed to relatively stable property of PtPDs in the cell without light irradiation, and decreased clearance by cells. The cytotoxicity of the PtPDs was measured, as shown in Fig. 2b and Figure S5. Cisplatin (Cis-Pt) exhibited dose-dependent inhibition of MB49 cancer cell growth, while PtPDs showed only slightly dark cytotoxicity. As expected, NIR light irradiation enhanced the cytotoxicity of PtPDs remarkably. The half maximal inhibitory concentration (IC_50_) values of PtPDs + L against BC cells were respectively 10.4, 43.8 and 4.2 times lower than those of Cis-Pt, PtPDs, and PDs + L, demonstrating the benefit of combining PTT and chemotherapy.

The performance of PtPDs against BC cells was further confirmed by co-staining with green calcein-AM and red propidium iodide (PI). As shown in Fig. 2c and d, the green fluorescence covering the entire field of view indicated that PtPDs in the dark exhibited negligible toxicity to MB49 cells, whereas free Cis-Pt exhibited increased toxicity. However, the combination of treatment with PtPDs and NIR light irradiation killed almost all MB49 cells, which was more effective than the Cis-Pt or PDs + L groups. In addition, we detected intracellular levels of GSH in PtPDs-treated cells. Generally, Pt-based drugs are easily inactivated by abundant thiol-containing GSH within tumor cells. As expected, PtPDs reduced intracellular GSH levels under NIR light irradiation to levels significantly below those of the other groups (Figure S6). In this case, PtPDs-mediated photochemotherapy dramatically induced the depletion of GSH, which greatly improved the therapeutic efficiency of Cis-Pt.

It is well known that chemotherapy, phototherapy, and radiotherapy elicited innate and adaptive immune responses by inducing ICD in cancer cell [43–45]. Tumor-associated antigens, proinflammatory cytokines and damage-associated molecular patterns (DAMPs) in such a process are able to recruit and activate immune effector cells for further immunotherapy [46, 47]. Thus, we investigated calreticulin (CRT) exposure, high mobility group box 1 protein (HMGB1) levels, and adenosine 5’-triphosphate (ATP) release in vitro following PtPDs-based photochemotherapy. As shown in Fig. 2e and Figure S7, PDs induced moderate CRT up-expression on the surface of MB49 cells upon NIR irradiation. However, PtPDs-mediated photochemotherapy remarkably elicited CRT expression, which was higher than that of the other groups. Similarly, PtPDs-mediated photochemotherapy induced the highest percentage of HMGB1 (41.3 ng/mL), while releasing the highest ATP level (38.9 nM) (Figure S8). It was also found that under NIR light irradiation, MB49 cancer cells treated with PtPDs + L greatly promoted in vitro BMDC maturation (17.8%) compared to any other single treatment (Fig. 2f and Figure S9). Correspondingly, DCs activated by PtPDs + L secreted the highest levels of TNF-α and IL-6 under NIR light irradiation (Fig. 2 g and Figure S10). Collectively, photochemotherapy based on PtPDs enhanced the ICD effect of Cis-Pt in vivo, providing promising vaccine-like benefits to anti-tumor therapy.

**Fig. 3:**
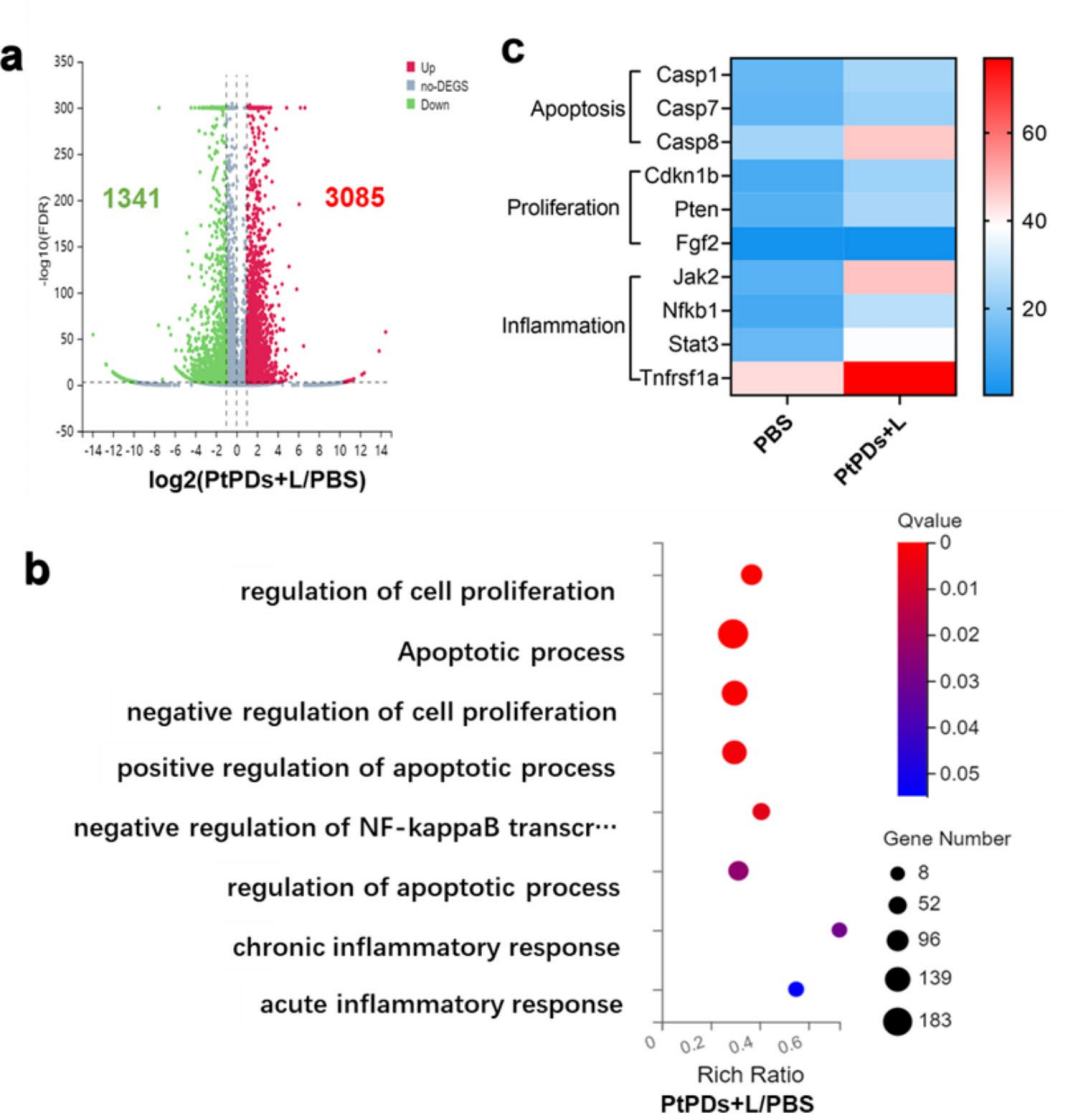
Differentially expressed genes (DEGs) analysis by RNA-Seq. **(A)** Volcano plot showing DEGs in MB49 cells between the PtPDs + L and PBS groups; DEGs: Differentially expressed genes. A red gene represents an upregulated gene, whereas a blue gene represents a downregulated gene (log 2 fold change (FC) ≤-1 and False Discovery Rate (FDR) ≤ 0.001). **(B)** Gene ontology (GO) enrichment analysis of DEGs; Qvalue ≤ 0.05 is considered a significant difference. **(C)** Expression heatmap of significant DEGs, which were laid out and classified by gene function using the NCBI NR database

To investigate PtPD-based photochemotherapy-mediated transcriptional regulation, cDNA libraries of MB49 cells were constructed for RNA-seq. As shown in Fig. 3, differentially expressed genes were investigated. In total, 3085 upregulated and 1341 downregulated DEGs were identified (log2 fold change ≥ 1 and FDR < 0.001) between the PtPDs + L and PBS groups. The associated DEGs are presented in a volcano plot (Fig. 3a), and they were enriched in the cell proliferation, inflammation, and apoptosis processes (Fig. 3b). Genetic profiling revealed that PtPDs + L upregulated genes that inhibit cell proliferation (PTEN and CDKN1b), apoptosis-related genes (Casp1, Casp7, and Casp8), and proinflammation (Tnfrsf1a, Nfkb1, Jak2, and Stat3), and downregulated genes that promote cell proliferation (Fgf2) when compared with the PBS group (Fig. 3c).

**Fig. 4:**
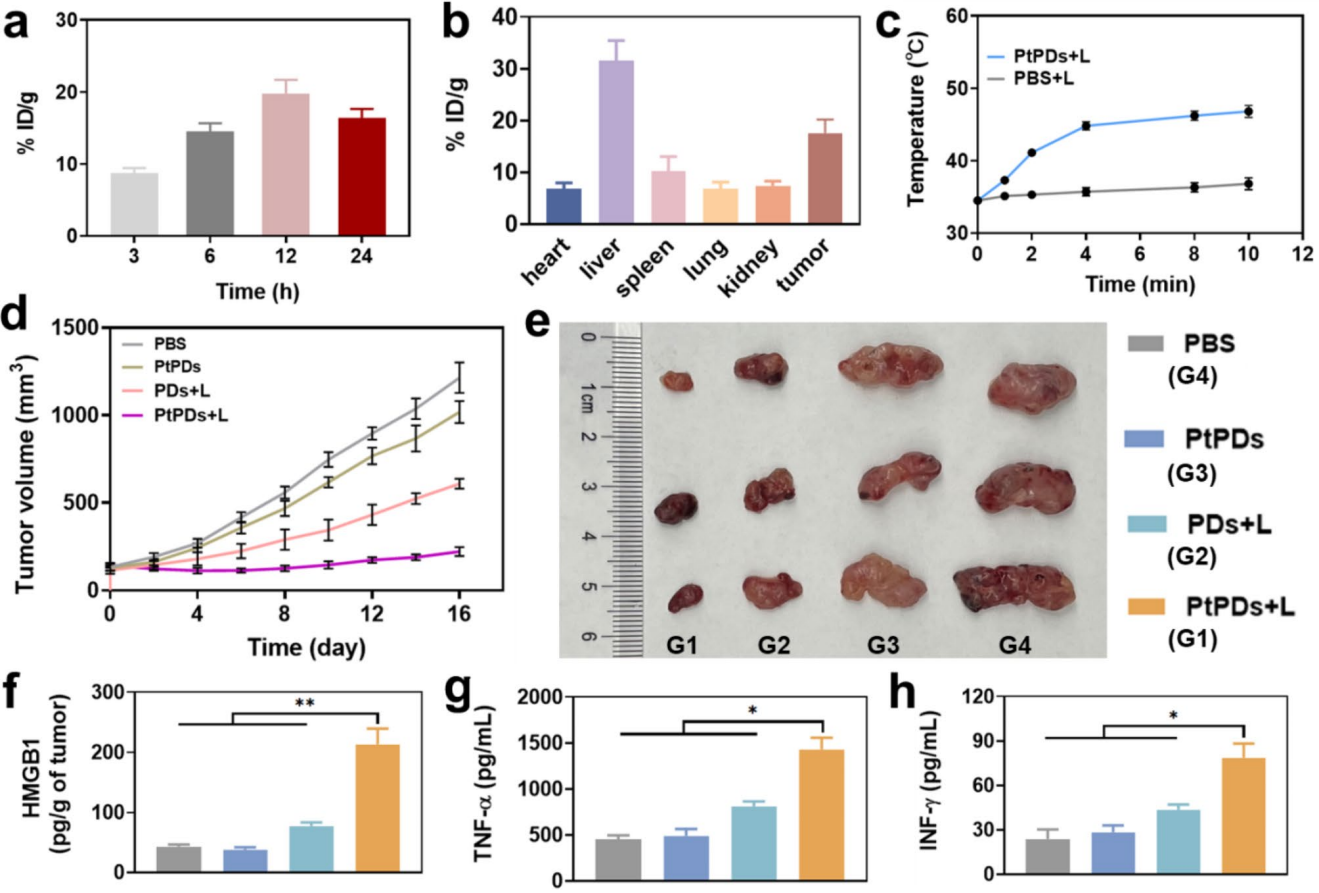
Biodistribution and photochemotherapy in vivo. **(a)** Accumulation of PtPDs in MB49 tumor was determined at different times. **(b)** Biodistribution of PtPDs in MB49 tumor-bearing mice was determined by ICP-MS. The major organs were extracted to evaluate the biodistribution of PtPDs 24 h after administration. **(c)** Photothermal capability of PtPDs at the tumor site. **(d)** Tumor volume and **(e)** Representative photographs of treated MB49-tumor bearing mice at day 16. **(f)** Intratumor HMGB1, **(g)** TNF-α, and **(h)** IFN-γ levels. All results represent the mean ± SD (n = 3), *p < 0.05, **p < 0.01

To determine the optimal timing of NIR light irradiation in vivo, we investigated the biodistribution of PtPDs in MB49 tumor-bearing mice by detecting the Pt content of tumor tissue at 3, 6, 12, and 24 h after intravenous administration. Pt accumulation at the tumor site increased over time, peaking 12 h after injection, and then decreased 24 h later (Fig. 4a). The higher accumulation of PtPDs in tumors may be associated with the prolonged circulation time (Figure S11). Thus, we determined that 12 h was the optimal time for further experiments. The PtPDs were found to exceed the size limit that could be cleared by the kidney (5 nm). The endothelial system is believed to be the primary pathway for the clearance of nanoparticles. As a result, the PtPDs accumulated in the liver (Fig. 4b), which was consistent with the biodistribution of most reported nanoparticles [7, 20, 23]. When the tumor volume reached approximately 100 mm^3^, the photothermal effect of PtPDs at the tumor site was evaluated 12 h after administration (Fig. 4c and Figure S12). The temperature of the PtPDs groups under 808 nm NIR irradiation (0.8 W/cm, 10 min) increased to 46.8 °C for efficient PTT compared with the PBS groups.

Based on these findings, we investigated the therapeutic efficacy of PtPDs-based photochemotherapy. We randomly divided the MB49 tumor-bearing mice into four groups: PBS, PtPDs, PDs + L, and PtPDs + L. Mice in the PDs + L and PtPDs + L groups were exposed to 808 nm NIR irradiation 12 h after intravenous administration. The tumors were collected on day 16. As shown in Fig. 4d and e, mice treated with PtPDs without light irradiation and PDs with light irradiation achieved delayed tumor growth, demonstrating that the curative effects of chemotherapy and PTT were insufficient on their own. Importantly, combination treatment involving PtPDs with light irradiation showed a superior tumor ablation effect, which notably outperformed the corresponding single therapeutic effect of the chemotherapy and PTT groups. Tumor growth inhibition was 16.2%, 50.2%, and 82.5% in the PtPDs, PDs + L, and PtPDs + L groups, respectively (Figure S13). The high therapeutic efficacy of PtPDs-mediated photochemotherapy is attributed to NIR light-induced PTT and Pt ion release, which triggers a cascade of tumor cell death. Combination therapy has been shown to effectively improve the efficacy of tumor treatment, and immunotherapy is a common approach used in combination therapy. This combination therapy approach is helpful in achieving a comprehensive treatment of tumors. To further explore the ICD effect and anti-tumor immune response in PtPDs-based photochemotherapy, we detected HMGB1 and pro-inflammatory factor release at the tumor site in each group. As expected, the PtPDs + L group showed the greatest release of HMGB1 (Fig. 4f), with higher levels of TNF-α, IFN-γ, and IL-6 than the other groups (Fig. 4 g, h, and Figure S14). These findings suggest that PtPDs-mediated photochemotherapy efficiently evoked stronger ICD effects and might stimulate a systematic anti-tumor immune response in combination with a checkpoint inhibitor.

**Fig. 5:**
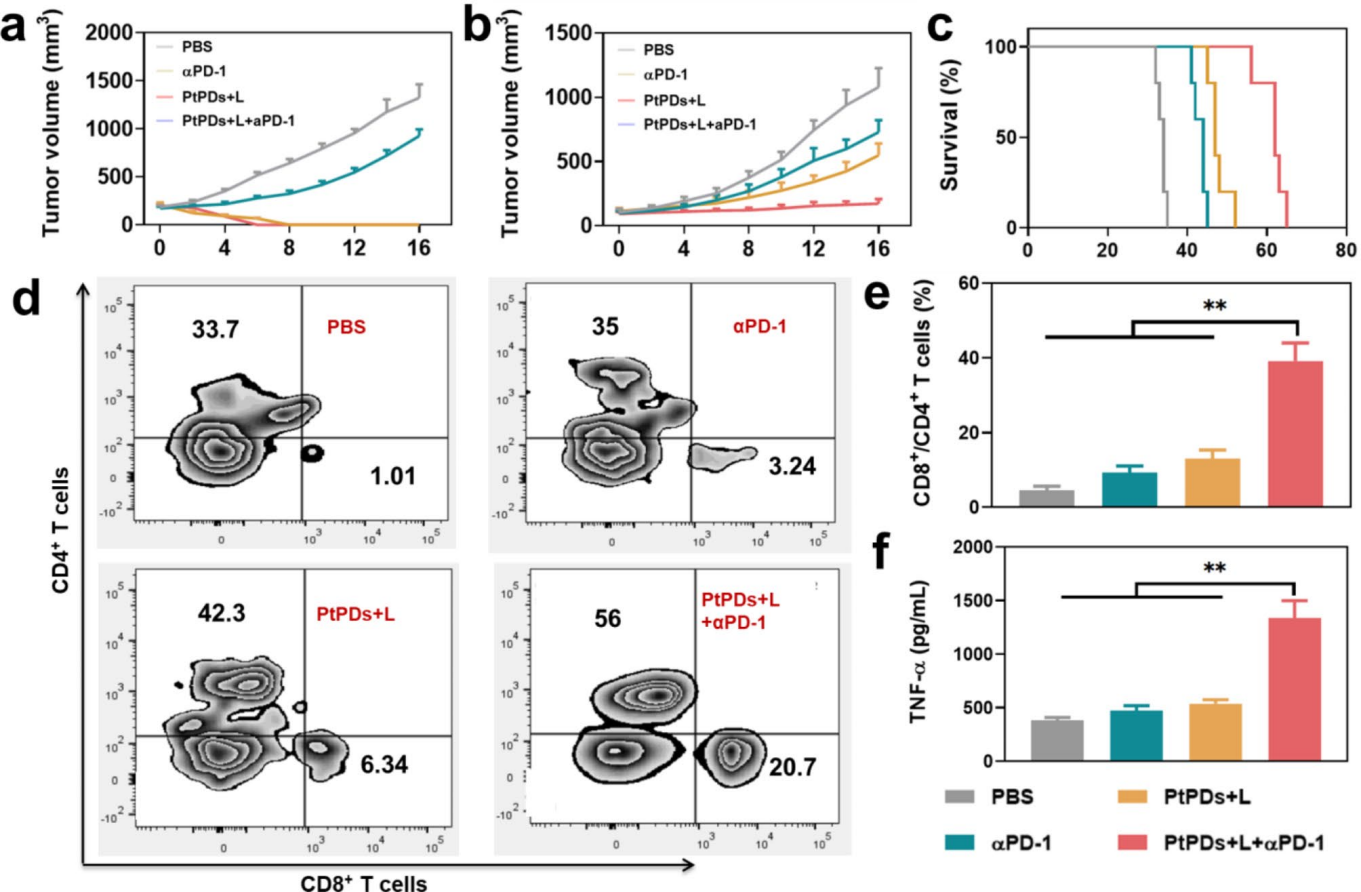
Anti-tumor therapeutic effects of PtPDs in a bilateral MB49 tumor model. Tumor growth curves of **(a)** primary and **(b)** distant tumors (n = 5). **(c)** Survival curves after treatment. Rechallenged tumor growth curves in mice bearing 4T1 tumors following the indicated treatments (n = 5). **(d)** Representative flow cytometric plots of intratumoral infiltration of cytotoxic CD8^+^ T cells (CD45^+^CD3^+^CD8^+^, gated on CD45^+^ T cells) and CD4^+^ T cells (CD45^+^CD3^+^CD4^+^, gated on CD45^+^ T cells), and **(e)** ratio of CD8^+^/CD4^+^ T cell in distant tumor tissues isolated at fifth day post-treatment (n = 5). d) Secretion levels of TNF-α in serum after various treatments. *p < 0.05, **p < 0.01

In light of their strong immune response, we developed a bilateral MB49 tumor model to evaluate the abscopal effect of PtPDs with the assistance of a PD-1 checkpoint blockade. Mice were assigned to four groups: PBS, αPD-1, PtPDs + L, and PtPDs + L + αPD-1. For the groups containing PtPDs, tumors on the left (primary tumors) were irradiated with NIR light, whereas tumors on the right (distant tumors) were not (Fig. 5b, c and Figure S15). PtPDs + L without NIR light irradiation dramatically ablated the primary tumor while exhibiting considerable inhibition of distant tumor growth. In contrast, αPD-1 alone did not markedly reduce the rapid growth of distant tumors. Importantly, combined with αPD-1, PtPDs + L under NIR light irradiation completely removed the primary tumor, but it also delayed the growth of distant tumors more effectively. Correspondingly, mice challenged with tumor cells survived until day 63 after PtPDs-based chemo-photoimmunotherapy (Fig. 5c), which may be attributed to the combined anti-tumoral immune response. Furthermore, intratumoral infiltrating immune cells in distant tumors were analyzed to demonstrate the systemic immune response. The percentage of cytotoxic CD8^+^ T lymphocytes (CTLs) and the population of CD8^+^/CD4^+^ T cells were significantly higher in the PtPDs + L + αPD-1 group (Fig. 5d-f and Figure S16) than in the other groups. Consistently, the highest levels of serum proinflammatory cytokines demonstrated systemic anti-tumor immunity (Figure S16).

**Fig. 6:**
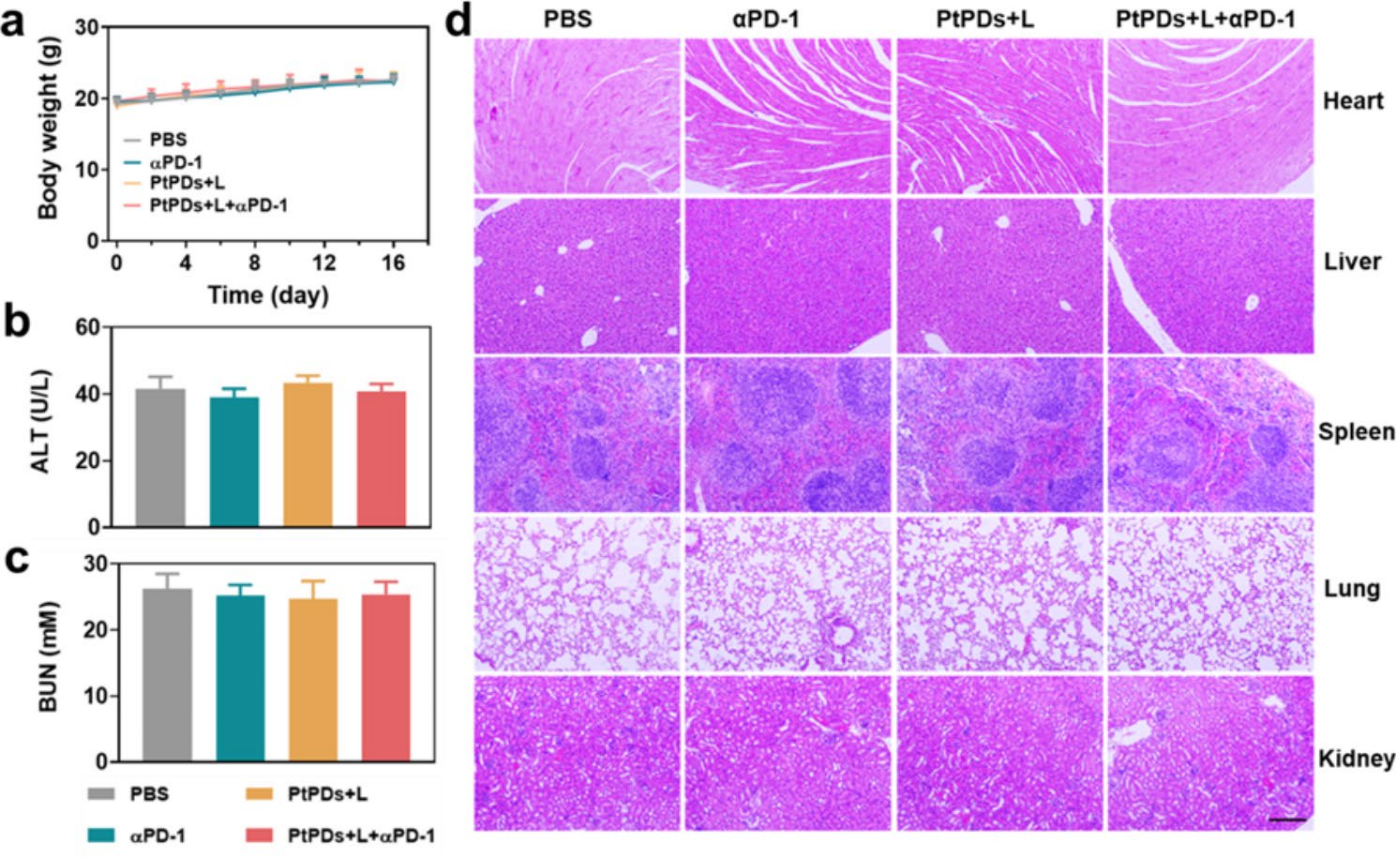
Biosafety of Pt-based chemotherapy. **(a)** Body weight for 16 days. Serum biochemistry indicators including **(b)** ALT, and **(c)** BUN for each treatment group at day16. All results represent the mean ± SD (n = 3). **(d)** Representative H&E images of main organs of each treatment group at day16. Scale bars indicate 50 μm

Because the biosafety of Pt-based chemotherapy is a significant risk in the clinic, we evaluated the safety profile of PtPDs-based chemo-photoimmunotherapy. Encouragingly, no significant pathology was observed in terms of body weight (Fig. 6a) and the levels of AST, ALT, BUN, and CRE (Fig. 6b and c, and Figure S17), which were consistent with the histopathology of the liver, spleen, kidney, lung, and heart in the mice of the αPD-1, PtPDs + L, and PtPDs + L + αPD-1 groups, and showed no significant difference when compared with the control group (Fig. 6d), demonstrating the inappreciable systemic toxicity of PtPDs-based chemo-photoimmunotherapy of BC.

## Conclusions

In summary, we developed self-polymerized platinum (II)-polydopamine nanocomplexes (PtPDs) with good photothermal capability to achieve efficient and safe chemo-photoimmunotherapy for BC. PtPDs were highly sensitive to the combined reductive tumor microenvironment and NIR light irradiation, leading to the degradation and controlled release of Pt ions. Consequently, PtPDs exhibited efficient photochemotherapy of BC in vitro and in vivo, strengthening ICD effects. When combined with immunotherapy, PtPD-based photochemotherapy elicits a systemic immune response to greatly inhibit primary and distant tumor growth with minimal adverse off-target effects. Notably, such a Pt-based nanomedicine with a simple structure has the potential to achieve efficient combined management of BC, particularly for advanced tumors. Collectively, the present work suggests the introduction of a versatile strategy via metal-dopamine self-polymerization for the precise delivery of other metal-based chemotherapeutic drugs in an efficient and safe manner.

## Methods

### Preparation and characterization of PtPDs

Cisplatin (400 mg, 1.33 mmol) and AgNO_3_ (406 mg, 2.39 mmol) were mixed in 10 mL of water [17, 35]. The pH was adjusted to 2 using a diluted HNO_3_ solution, and the mixture was stirred overnight in the dark at 70 °C and cooled overnight. The precipitate was then filtered to obtain activated cisplatin, using a 0.22 μm syringe filter. Subsequently, 100 mg of activated cisplatin and 300 mg of dopamine hydrochloride were dissolved in 200 mL of water and stirred for 1 h, after which the mixture was adjusted to pH 8 and stirred for another 2 h at 30 °C. The mixture was dialysis in deionized water to remove any unreacted agents, followed by lyophilized to obtained PtPDs.

### GSH and light-responsive drug release of PtPDs

To analyze drug release, 5 mg of PtPDs were placed in a dialysis bag with a molecular weight cut-off (MWCO) of 3500 Da. The bag was then added to 10 mL of phosphate-buffered saline (PBS) with or without 10 mM GSH. The samples were kept in darkness or irradiated with 808 nm near-infrared (NIR) light at a power density of 0.5 W/cm^2^ for 10 min. After that, 1 mL of the solution was collected, and the released Pt in the supernatant was measured at predetermined time points. The Pt was quantified using inductively coupled plasma mass spectrometry (ICP-MS). The GSH concentration in the supernatant was determined using Ellman’s reagent.

### Cellular uptake of PtPDs

The MB49 murine BC cell line was obtained from the American Type Culture Collection (ATCC, Manassas, VA) and cultured in RPMI 1640 medium with 10%(v/v) fetal bovine serum (FBS) and 1% antibiotic/antimycotic solution at 37 °C in an incubator with 5% CO_2_.

To determine the cellular internalization, MB49 cells were seeded into 24-well plates (5 × 10^4^ cells/well) and incubated overnight. After exposure to Cy5-labeled PtPDs for different durations at a final concentration of 10 µg/mL, cells were rinsed and stained with DAPI. All samples were observed under a fluorescence microscope. The cellular uptake of PtPDs was further quantified using ICP-MS.

### In vitro anti-tumoral effects of PtPDs

Cell viability was measured using the SRB assay. 5 × 10^3^ MB49 cells were plated into 96-well plates and incubated overnight. The cells were then treated with different formulations (Cis-Pt, PtPDs, PDs + L, and PtPDs + L). Cells were exposed to NIR light (808 nm, 0.5 W/cm^2^, 10 min) after incubation with the material for 12 h. After 24 h, the optical density (OD) at a wavelength of 570 nm was measured to calculate cell viability and IC_50_. For the live-dead cell assay, a 24-well plate was seeded with 3 × 10^4^ cells per well and cultured overnight. After treatment with the same group as the cell viability experiment for 24 h, all cells were observed by fluorescence microscopy after staining with Calcein AM-PI.

### In vitro GSH depletion of PtPDs

To detect intracellular GSH depletion, MB49 cells were treated with various formulations (PBS, Cis-Pt, PDs, PtPDs, and PtPDs + L) for 24 h. The intracellular GSH content was determined using a Micro Reduced GSH Assay Kit. The total glutathione concentration in the cells was calculated as ng/mg protein.

### In vitro ICD examination of PtPDs

The exposure of calreticulin (CRT) of cells treated with PtPDs was detected by flow cytometry. 2 × 10^5^ MB49 cells were plated into 6-well plates, and overnight cultivation was carried out. Then cells were treated with a series of formulations (PBS, Cis-Pt, PDs + L, PtPDs, PtPDs + L) for 24 h. Radiation (0.5 W/cm^2^, 10 min) of 808 nm light was applied to the PtPDs + L and PDs + L groups after 12 h incubation. After washing with cold PBS a 30-minute staining with Alexa Fluor 488-CRT antibody was performed, and the result was analyzed using flow cytometry (Beijing Challen Biotechnology Co., Ltd, FongCyte). The levels of chromatin-binding protein high mobility group B1 (HMGB1) and adenosine triphosphate (ATP) released by the treated MB49 cells were detected using HMGB1 and ATP assay kits.

### In vitro dendritic cell (DC) maturation of PtPDs

For *the in vitro* DC maturation experiment, 2 × 10^5^ MB49 cells were plated into 6-well plates, and overnight cultivation was carried out. Subsequently the samples were treated with formulations (PBS, Cis-Pt, PDs, PtPDs, PtPDs + L) for 24 h and then co-cultured with bone marrow-derived dendritic cells (BMDCs) at a ratio of 1:1. After collection, non-adherent BMDCs were washed and stained with FITC-CD11c and Cy5.5-CD40 monoclonal antibodies. The maturation level of the BMDCs was determined using flow cytometry. The concentrations of proinflammatory cytokines (TNF-α, IL-6, and IFN-γ) in the supernatant were detected using an ELISA kit.

### Transcriptome sequencing analysis

MB49 tumor cells were collected and resuspended in PBS. After centrifugation for 5 min at 400 g, the supernatants were discarded. The cells were then transferred to liquid nitrogen. Total RNA extraction, mRNA library construction, transcriptome sequencing, and gene expression analysis were performed at BGI (Shenzhen, China). Differentially expressed genes (DEGs) analysis for mRNA was performed using the PossionDis algorithm with |log2 fold change (FC)|≥1 and false discovery rate (FDR) ≤ 0.001. Our study classified DEGs based on gene ontology (GO) annotation and enriched GO functionality using phyper, a function in the R programming language. FDR correction was performed on the Pvalue to obtain the Qvalue, and a function with Qvalue ≤ 0.05 was considered significantly enriched.

### Biodistribution of PtPDs in vivo

Female C57BL/6J wild-type mice (5–7 weeks) were obtained from Hunan SJA Laboratory Animal Company, Ltd. and acclimatized for one week before the experiments. Animal experiments were approved by the Ethics Committee and Institutional Animal Care and Use Committee of the South China University of Technology (Guangzhou, China). The MB49 BC model was established by injecting 1 × 10^6^ MB49 cells into the right flank of each mouse. The MB49 tumor-bearing mice were injected intravenously with PtPDs (20 mg/kg, n = 5) once the tumor volume reached approximately 100 mm^3^. Tumors were collected 3, 6, 12, and 24 h after injection. The Pt content in each group was determined by ICP-MS. The major organs were extracted to evaluate the biodistribution of PtPDs 24 h after administration.

### In vivo anti-tumor activity and ICD induction of PtPDs

When the volume of the MB49 tumor reached approximately 100 mm^3^, the tumor-bearing mice were randomly divided into five groups. The mice were intravenously injected with PBS, PtPDs, PDs + L, or PtPDs + L every 4 days for two doses (15 mg/kg, n = 6). In the light-treated group, mice were irradiated with NIR light (808 nm, 0.8 W/cm^2^, 10 min) 12 h after injection. The anti-tumor activity was evaluated by monitoring the tumor volume with a digital caliper every 2 days. The tumor volume was calculated. After 16 days of therapy, the mice were sacrificed, and the tumors were calculated. The HMGB1, TNF-α, IL-6, and IFN-γ level in tumor was determined using an ELISA kit.

### Anti-tumor activity, immune response, and safety profile of PtPD-based photochemo-immunotherapy

To develop the bilateral tumor model, MB49 cells were injected subcutaneously into the left flank (primary tumor) and right flank (distant tumor). When the tumor volume reached approximately 100 mm^3^, mice were randomly assigned to four groups: PBS, αPD-1, PtPDs + L, and PtPDs + L + αPD-1. The primary tumors were injected intratumorally with PBS or PtPDs on day 1. For the group containing PtPDs, the left tumors were subjected to NIR light irradiation (808 nm, 0.8 W/cm^2^, 10 min), while the right tumors received no treatment. Then, the αPD-1 and PtPDs + L + αPD-1 mice were injected intravenously with αPD-L1 antibody on days 2, 5, and 8 post-injection at a dose of 100 µg/mouse. Body weight and sizes of primary and distant tumors were recorded every two days. Mouse serum was collected for TNF-α, IFN-γ, and IL-6 measurement on the ninth day after injection. Distant tumors were collected and processed to determine T cell infiltration by flow cytometry. After 16 days of therapy, the mice were sacrificed and the lung, liver, heart, kidney, and spleen were collected for H&E staining. The levels of serum alanine aminotransferase (ALT), aspartate transaminase (AST), blood urea nitrogen (BUN), and creatinine (CRE) were evaluated using Coulter LX2D instrumentation (Beckman, Brea, CA, USA).

## Electronic supplementary material

Below is the link to the electronic supplementary material.

Supplementary Material 1

## Data Availability

The datasets used and/or analyzed during the current study are available from the corresponding author on reasonable request.

## Abbreviations

ICD: Immunogenic cell death
NIR: Near-infrared
BC: Bladder cancer
PDs: Polydopamine
PtPDs: Platinum (II)-polydopamine nanocomplexes
CLTs: Cytotoxic CD8^+^ T lymphocytes
PBS: Phosphate-buffered saline. ICP-MS:inductively coupled plasma mass spectrometry. FBS:fetal bovine serum
OD: Optical density
CRT: Calreticulin
ATP: Adenosine triphosphate
HMGB1: High mobility group B1
DC: Dendritic cell
BMDCs: Bone marrow-derived dendritic cells
DEGs: Differentially expressed genes
GO: Gene ontology
ALT: Alanine aminotransferase
AST: Aspartate transaminase
BUN: Blood urea nitrogen blood urea nitrogen blood urea nitrogen
CRE: Creatinine
