# Supplementary material for: Self-polymerized platinum (II)-Polydopamine nanomedicines for photo-chemotherapy of bladder Cancer favoring antitumor immune responses
Source: J Nanobiotechnology. 2023 Jul 22;21:235. doi: 10.1186/s12951-023-01993-1 (PMC10362689; doi:10.1186/s12951-023-01993-1)

**Self-polymerized Platinum (II)-Polydopamine Nanomedicines for Photo-chemotherapy of Bladder Cancer Favoring Antitumor Immune Responses**

Ren Mo ^1^ † *, Jianati Dawulieti ^2, 3,^ †, Ning Chi ^1,^ †, Ziping Wu ^4^, ZhiZhong Yun ^1^, JianJun Du ^1^, XinHua Li ^1^, JunFeng Liu ^1^, Xiaochun Xie ^4^, Kai Xiao ^4^, Fangman Chen ^5^ *, Dan Shao ^4^, KeWei Ma ^1, 6^ *

**1. Materials and agents**

Cisplatin and dopamine hydrochloride were purchased from Adamas. AgNO_3_ was purchased from Chengdu Kelong Chemical Co., Ltd. HNO_3_ solution was purchased from. PBS and ATP assay kits were purchased from Beyotime Biotechnology. RPMI 1640 medium, fetal bovine serum (FBS), and 0.25% trypsin-EDTA was purchased from Gibco Co., Ltd. (Carlsbad, CA, U.S.A.). Micro Reduced GSH Assay Kit and Living/Dead cell double staining kit (Calcein-AM/PI) was purchased from solarbio. Calreticulin Polyclonal Antibody, ALEXA FLUOR® 488 Conjugated was purchased from Bioss Antibodies, FITC Anti-Mouse CD11c Monoclonal Antibody and Cy5.5-CD40 monoclonal antibodies were purchased from. Mouse HMGB-1 (High Mobility Group Pritein B1) ELISA Kit, Mouse TNF-α (Tumor Necrosis Factor Alpha) ELISA Kit，IFN-γ (Interferon γ) ELISA Kit and Mouse IL-6 (Interleukin 6) ELISA Kit were purchased from Elabscience. GM-CSF and IL-4 were purchased from PeproTech, Inc. Anti-CD11c-FITC, anti-CD86-PE, and anti-CD80-APC were purchased from BD (Shanghai, China). Anti-CD45-BV605, Anti-CD4-FITC, anti-CD3-PerCP/cy5.5, and anti-CD8a-APCwere purchased from BioLegend, Inc. The anti-mouse PD-1 mAb (αPD-1) was purchased from Bio X Cell (Lebanon, NH, USA).

**2. Characterization**

The morphologies of the MSNs were characterized with a JEM-2100F transmission electron microscope (TEM, JEOL, Ltd., Japan) and a scanning electron microscope (SEM, FEI Quanta 200F). The hydrodynamic diameter and zeta potential of the NPs were characterized with a Nano-ZS 90 Nanosizer (Malvern Instruments Ltd., Worcestershire, UK). UV-vis adsorption spectra were recorded on a U-3310 spectrophotometer (Hitachi, Japan). Specific surface area and pore size distributions were evaluated and calculated by the Brunauer-Emmett-Teller (BET) and Barrett-Joyner-Halenda (BJH) methods. Stability experiments were performed by measuring nanoparticles in DMEM plus 10% FBS for 7 days using a Nano-ZS 90 Nanosizer.

**3. Statistical analysis**

Differences between groups were analyzed by Student’s test when comparing only two groups. Differences among more than two groups were analyzed by one-way analysis of variance, and the Bonferroni post hoc test was used to analyze differences between any two groups. *P < 0.05 was considered significantly difference


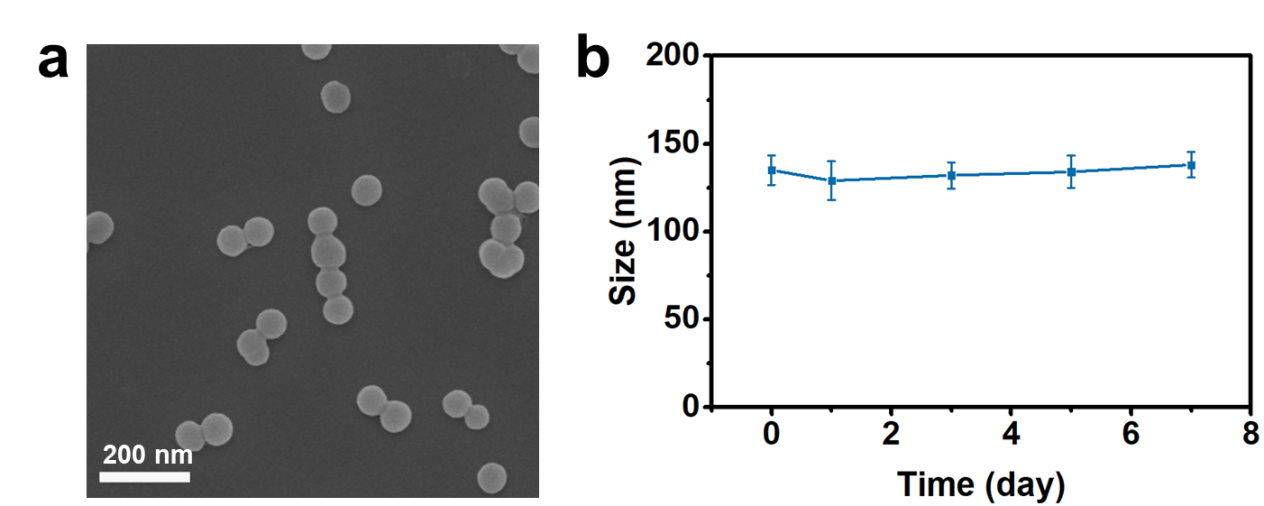


**Figure S1**. Characterization of PtPDs. a) SEM image, b) stability of PtPDs in cell culture media.


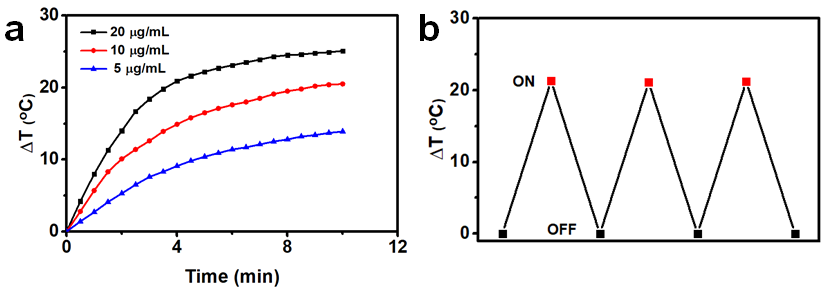


**Figure S2**. Photothermal performance of PtPDs. a) The photothermal capability of PtPDs at different Fe concertration (0.5 W/cm). b) The temperature of PtPDs was recorded when we irradiated photo-thermal material by 808 nm laser repeatedly.


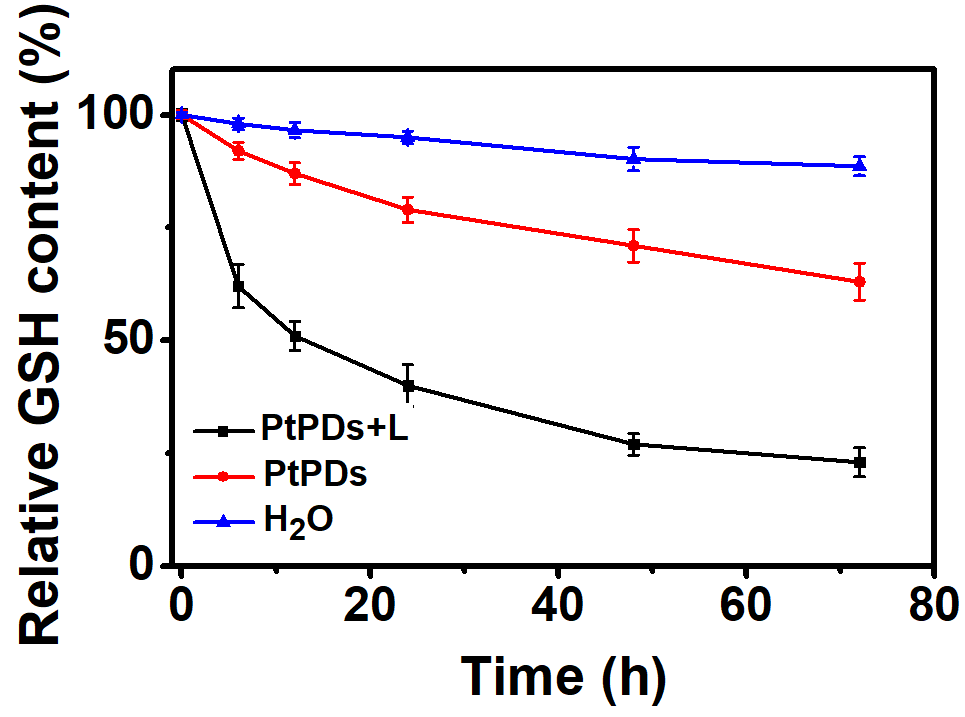


**Figure S3**. The level of GSH was measured in PtPDs with or without NIR irradiation.


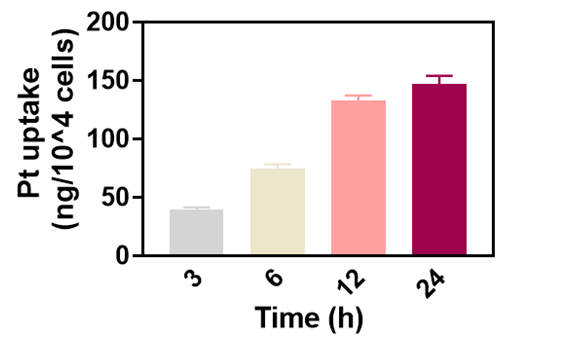


**Figure S4**. Quantitative analysis of intracellular Pt content as determined by ICP-MS.


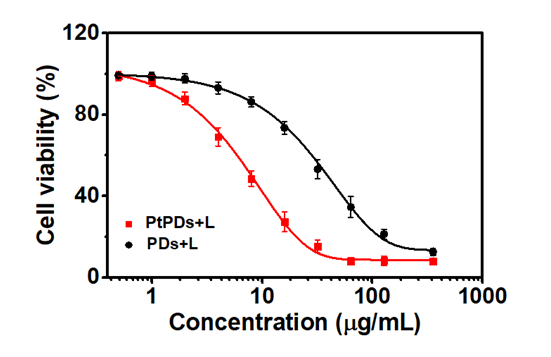


**Figure S5**. Cell viability of MB49 cells after incubation for 24 h with 808 nm laser exposure (0.5 W/cm, 10 min).


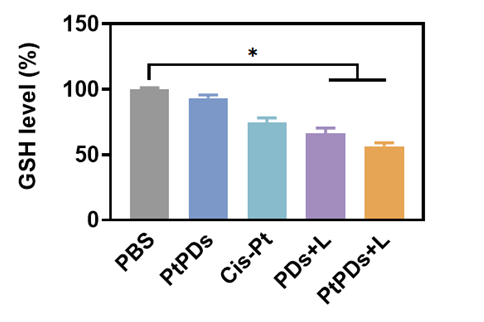


**Figure S6**. Intracellular GSH level in MB49 cells treated with different formulation at 10 μg/mL Cis-Pt or corresponding materials (60 μg/mL). *p < 0.05.


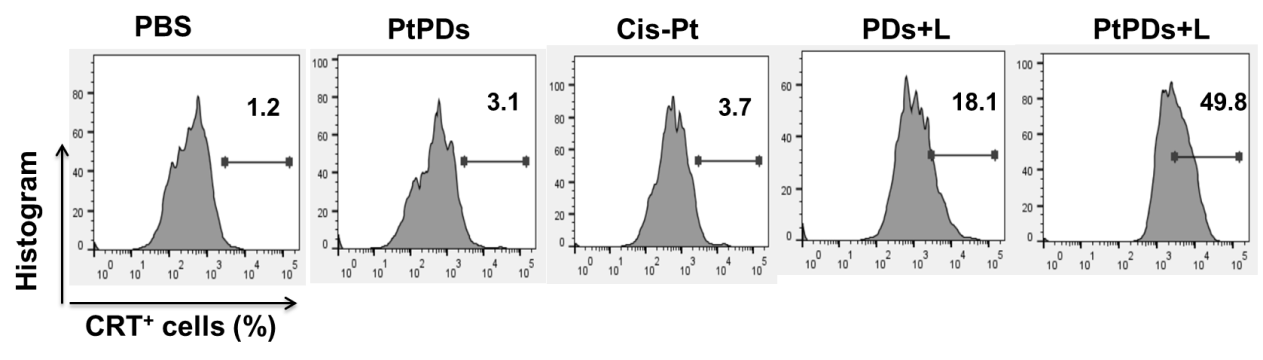


**Figure S7**. Flow cytometric examination of CRT exposure on the surface of MB49 tumor cells after various treatments.


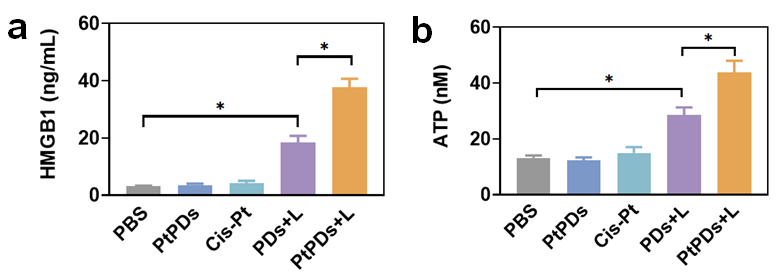


**Figure S8**. Efficient induced Immunogenic cells death of the tumor cells by PtPDs. Quantitative examination of released HMGB1 and c) ATP secretion from MB49 tumor cells after various treatments at 10 μg/mL Cis-Pt or corresponding materials (60 μg/mL) for 24 h. *p < 0.05.


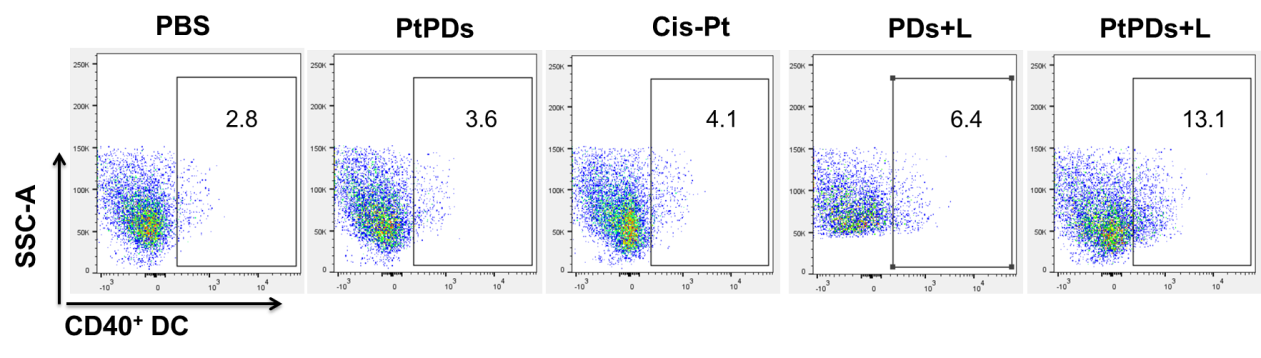


**Figure S9**. Quantification of matured BMDCs (gated on CD11c^+^ and CD40^+^ cells) was analyzed by flow cytometry after different treatments for 24 h.


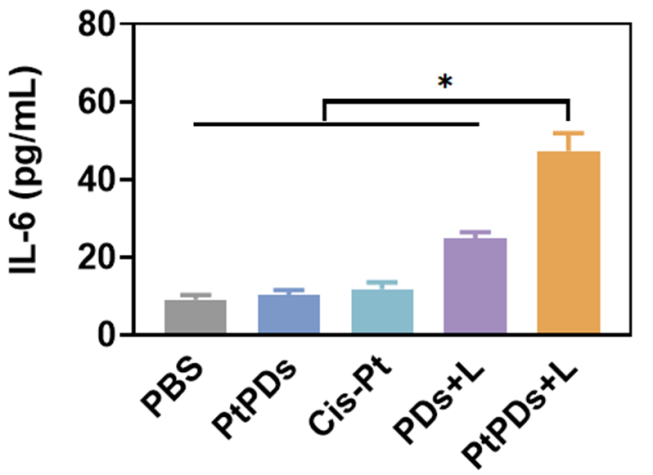


**Figure S10**. The secretion levels of IL-6 in matured BMDCs suspensions. *p < 0.05. Data are presented as means ± SD. (n = 3).


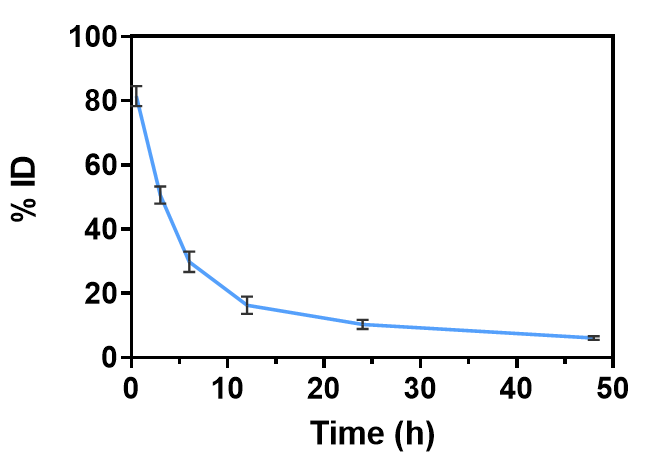


**Figure S11**. Blood circulation time profile of PtPDs in MB49 tumor bearing mice after i. v. injection.


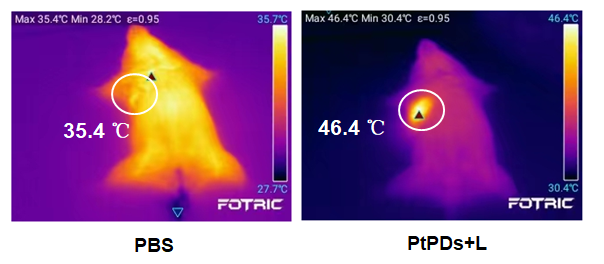


**Figure S12**. *In vivo* photothermal effects of PBS and PtPDs upon light irradiation (0.8 W/cm, 10 min).


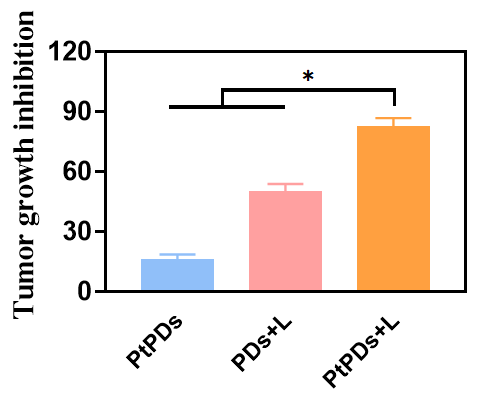


**Figure S13**. The tumor growth inhibition in MB49 tumor bearing mice after various formulations treated. *p < 0.05.


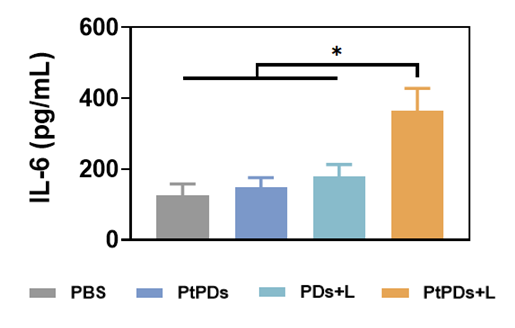


**Figure S14**. ELISA analysis induced secretion of proinflammatory cytokine levels of IL-6 after various treatments. *p < 0.05.


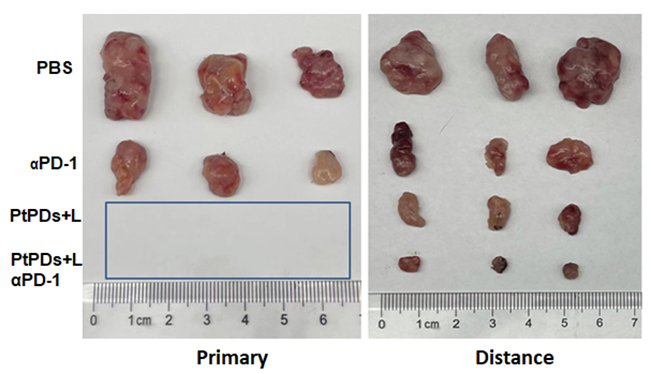


**Figure S15**. Photographs of treated MB49-tumor bearing mice over 16 days.


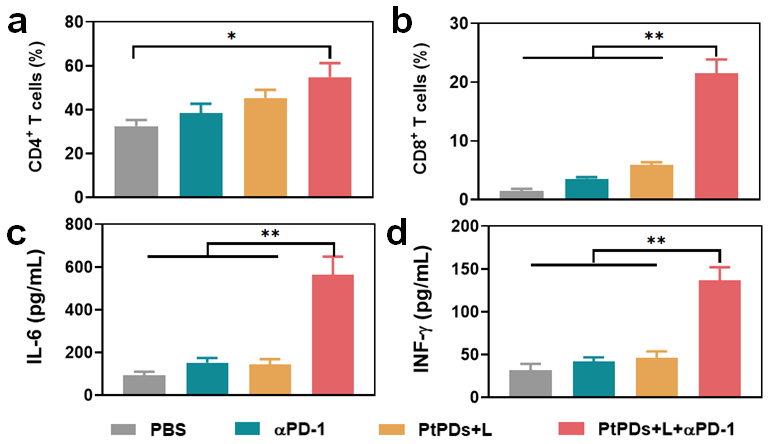


**Figure S16**. Antitumor immunity of PtPDs in a bilateral MB49 tumor model. quantitative data of intratumoral infiltration of cytotoxic CD4^+^ T cells (CD45^+^CD3^+^CD4^+^, gated on CD45^+^ T cells), and b) CD8^+^ T cells (CD45^+^CD3^+^CD8^+^, gated on CD45^+^ T cells) in distant tumor tissues isolated at 5th post-treatment (n = 5). Secretion levels of c) IL-6 and d) IFN-γ in serum after various treatments. *p < 0.05, **p < 0.01.


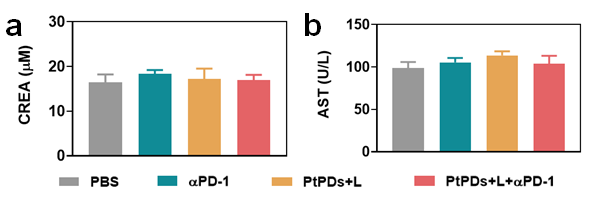


**Figure S17**. Serum biochemistry indicators of a) creatinine (CREA) and b) aspartate aminotransferase (AST), for each treatment group at 16 days.

Table S1. Characterization of PDs and PtPDs.


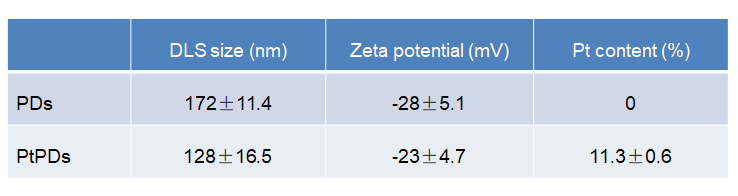

Supplement: Supplementary file 1 — Supplementary Material 1 [file 12951_2023_1993_MOESM1_ESM.docx]
